# Aptamer-based cocaine assay using a nanohybrid composed of ZnS/Ag_2_Se quantum dots, graphene oxide and gold nanoparticles as a fluorescent probe

**DOI:** 10.1007/s00604-019-4101-6

**Published:** 2020-01-08

**Authors:** Oluwasesan Adegoke, Magda A. Pereira-Barros, Svetlana Zolotovskaya, Amin Abdolvand, Niamh Nic Daeid

**Affiliations:** 1 Leverhulme Research Centre for Forensic Science, University of Dundee, Dundee, DD1 4GH United Kingdom; 2 Materials Science & Engineering Research Cluster, School of Science & Engineering, University of Dundee, Dundee, DD1 4HN United Kingdom

**Keywords:** Nanohybrid, Graphene oxide, Quantum dots, Gold nanoparticle, Fluorescence

## Abstract

Authors report on a new fluoro-graphene-plasmonic nanohybrid aptamer-based fluorescent nanoprobe for cocaine. To construct the nanoprobe, newly synthesized glutathione-capped ZnS/Ag_2_Se quantum dots (QDs) were first conjugated to graphene oxide (GO) to form a QD-GO nanocomposite. The binding interaction resulted in a fluorescence turn-ON. Thereafter, cetyltrimethylammonium bromide (CTAB)-gold nanoparticles (AuNPs) were directly adsorbed on the QD-GO nanocomposite to form a novel QD-GO-CTAB-AuNP nanohybrid assembly that resulted in a fluorescence turn-OFF. Streptavidin (strep) was then adsorbed on the QDs-GO-CTAB-AuNP nanohybrid assembly which allowed binding to a biotinylated MNS 4.1 anticocaine DNA aptamer (B) receptor. The addition of cocaine into the strep-B-QDs-GO-CTAB-AuNP aptamer nanoprobe system aided affinity to the aptamer receptor and in turn turned on the fluorescence of the nanoprobe in a concentration-dependent manner. Under optimum experimental conditions, we found the strep-B-QD-GO-CTAB-AuNP to be far superior in its sensitivity to cocaine than the tested strep-B-QDs (no GO and CTAB-AuNPs), strep-B-QD-CTAB-AuNP (no GO) and strep-B-QD-GO (no CTAB-AuNP). In addition, the investigation of localized surface plasmon resonance (LSPR) amplified signal from tested plasmonic NPs shows that CTAB-AuNPs was far superior in amplifying the fluorescence signal of the nanoprobe. A detection limit of 4.6 nM (1.56 ng.mL^−1^), rapid response time (~2 min) and excellent selectivity against other drugs, substances and cocaine metabolites was achieved. The strep-B-QD-GO-CTAB-AuNP aptamer-based fluorescent nanoprobe was successfully applied for the determination of cocaine in seized adulterated cocaine samples.

**Electronic supplementary material:**

The online version of this article (10.1007/s00604-019-4101-6) contains supplementary material, which is available to authorized users.

**Graphical abstract:**
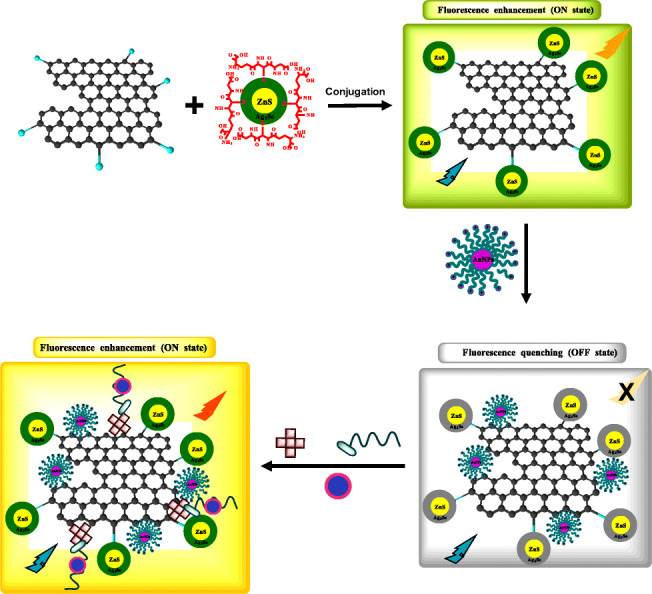
Schematic representation of the streptavidin-biotin-quantum dot-graphene oxide-cetyltrimethylammonium bromide-gold nanoparticle aptamer-based fluorescent nanoprobe for cocaine.

## Introduction

Abuse of cocaine remains a critical public health challenge due to the associated health risks connected with its illicit use. Organ damage, cardiovascular problems, insomnia, loss of appetite, violent behaviour, anxiety and paranoia are all documented examples of health-related issues associated with cocaine use [1]. Rapid, simple, selective, sensitive and inexpensive methods for cocaine detection are currently needed to meet the detection requirements (both presumptive and confirmatory) for efficient analysis. Traditional confirmatory chemical analysis for cocaine identification relies on complex separation techniques such as high-performance liquid chromatography (HPLC) and gas chromatography-mass spectrometry (GC-MS) [2]. Despite these techniques being able to achieve detection limit levels required for confirmatory identification of cocaine, practical drawbacks for real-life implementation such as reproducibility, throughput, mobility and cost, often leads to challenging decisions on the trade-offs associated with various aspects of the analytical device output if further miniaturization are desired for real-time, in-field and wearable applications [3]. To overcome the drawbacks of chromatographic techniques, aptamer-based nanoprobes have been investigated as alternative detection methods for cocaine identification [4].

Aptamers are single-stranded synthetic DNA or RNA oligonucleotide sequences that selectively bind to a target analyte via shape recognition [5]. Aptamers used in biosensing applications are nearly exclusively artificial and are evolved from a large random pool of oligonucleotide sequences through the process known as Systematic Evolution of Ligands by Exponential Enrichment (SELEX) [6]. To date, a variety of aptameric nanoprobes based on fluorescence transduction assays have been developed for cocaine [7–9]. However, there is still a continual need to develop more efficient probes to meet the growing demand of rapid and accurate testing of illicit drugs.

With the advent of new nano-based materials and components, more sophisticated soft-matter nanofabrication and targeted assembly techniques can lead to the development of prospective nanoprobes devices for illicit drugs by utilizing highly selective and sensitive biocompatible sensing elements. There is little doubt that nanoprobes based on the direct combination of nanoreporter elements and biorecognition units, that are capable of molecular binding events and on-spot transduction, are a far more efficient method for biorecognition unit utilization than traditional chemical-based assays. It is foreseen that the utilization of hybrid nanostructures in nanoprobe development [10] may pave the way to overcome the challenges associated with achieving global commercial success for nanobiosensing of illicit drugs.

In this work, we report on the development of a new fluorescent nanohybrid assembly nanoprobe platform for cocaine identification based on the use of an anticocaine aptamer as a bioreceptor and fluorescence technique as the transduction signal. ZnS/Ag_2_Se quantum dots (QDs), capped with _L_-glutathione (GSH), and a novel component for the nanoprobe platform was synthesized and used as a fluorescent-emitting nanoreporter element.

Graphene oxide (GO), one of the components of the nanohybrid assembly, is a 2D lattice of graphitic carbon atoms densely packed in a honeycomb like hexagonal pattern [11]. The large 2D aromatic structural surface of GO makes it an ideal platform for the adsorption of biomolecules, while its electrical properties and high surface-to-volume ratio are known to influence sensing signals [12]. Metallic gold nanoparticle (AuNP), another component of the nanohybrid assembly, is characterized by the unique absorption and scattering properties dominated by the localized surface plasmon resonance (LSPR) and is considered the most stable of the noble metal NP class [13]. Its resistance to surface oxidation and chemical inertness have attracted extensive use in nanotechnology applications [14]. Particularly, the LSPR signal of AuNPs has been known to amplify fluorescence signal of QDs, thereby leading to lower detection limits [15].

To construct the nanoprobe, GO was first conjugated to _L_-glutathione (GSH)-ZnS/Ag_2_Se core/shell QDs to form a QDs-GO nanocomposite which resulted in a fluorescence enhancement (“ON state”) process. Thereafter, plasmonic cetyltrimethylammonium bromide (CTAB)-AuNPs were adsorbed onto the QDs-GO nanocomposite to form a QDs-GO-CTAB-AuNP nanohybrid assembly which triggered a fluorescence quenching (“OFF state”) process. Streptavidin (Strep) protein was then adsorbed onto the QDs-GO-CTAB-AuNP nanohybrid to form a strep-QDs-GO-CTAB-AuNP nanohybrid assembly. The incorporation of a biotinylated anticocaine DNA aptamer (B) into the strep-QDs-GO-CTAB-AuNP nanohybrid system, ensured the binding affinity to the adsorbed streptavidin. Cocaine being added to the nanoprobe system and its subsequent binding to the aptamer receptor, triggered fluorescence enhancement of the strep-B-QDs-GO-CTAB-AuNP nanohybrid nanoprobe (“ON state”). The plasmonic CTAB-AuNP was found to amplify the fluorescence signal in the presence of detected cocaine concentration via localized surface plasmon resonance (LSPR)-enhanced fluorescence intensity. To the best of our knowledge, this is the first reported QDs-GO-CTAB-AuNP nanohybrid aptamer-based fluorescence nanoprobe for cocaine.

## Experimental

### Materials

Silver nitrate (AgNO_3_) (≥ 99%), sodium chloride (≥ 99%), potassium chloride (99.0–100.5%), acetaminophen (≥ 99.0%), d-amphetamine, (+) methamphetamine hydrochloride, sodium phosphate dibasic (≥ 99.0%), potassium phosphate dibasic (≥ 99.0%), benzocaine (≥ 99%), lidocaine hydrochloride (≥ 99%), cocaine hydrochloride (≥ 97.5%), (+) methamphetamine hydrochloride, phenacetin (≥ 98%), benzoylecgonine solution, ecgonine methyl ester solution, octadecene (90%), trioctylyphosphine oxide (99%), trioctylyphosphine (97%), hexadecylamine (90%), sulphur (99.999%), selenium (Se) (99.5 + %), oleic acid (99%), and H_2_O_2_ (30% *w*/w) in solution with stabilizer were purchased from Sigma Aldrich (https://www.sigmaaldrich.com/united-kingdom.html). Tris(hydroxymethyl) aminomethane (≥99.8%) was purchased from Formedium (https://www.formedium.com/). Levamisole HCl (99 + %), diltiazem (98%), CTAB (∽98%), MES (99 + %), gold (III) chloride trihydrate (HAuCl_4_.3H_2_O) (≥99.9%), potassium acetate (KAc) (≥99.0%), Thermo Scientific™ Pierce™ EDC Crosslinker, N-hydroxysuccinimide, sodium acetate (NaAc), graphite powder (99.9995%), diethylzinc, GSH (98 + %) and potassium permanganate (KMnO_4_) (99 + %) were purchased from Thermo Fisher (https://www.fishersci.co.uk/gb/en/home.html). The biotinylated MNS 4.1 anticocaine DNA aptamer oligonucleotide with the nucleic acid sequence:

BIOTEG-5ˈ- GGGAGACAAGGAAAATCCTTCAATGAAGTGGGTCGACA was synthesized and purified by Eurofins (https://www.eurofins.co.uk/). All other chemicals were used as received. A Milli-Q water system was used as the water source.

### Characterization

Fluorescence and UV/vis absorption measurements were carried out on a Cary Eclipse (Varian) spectrophotometer. Scanning electron microscopy (SEM) and energy dispersive X-ray (EDX) analysis were carried out using a JEOL JSM 7400F field emission SEM integrated with an Oxford Instruments Inca EDX spectrometer. Transmission electron microscopy (TEM) measurements were carried out using a JEOL JEM-1200EX operated at 80 kV. Zeta potential (ZP) and dynamic light scattering (DLS) analysis were carried out using Zetasizer Nano ZS series (ZEN3600, Malvern). The Raman spectra were collected using an in-house built microprobe system equipped with a continuous wave laser sources emitting at 633 nm, the Oriel MS257 monochromator fitted with the Andor Newton EMCCD detector TE-cooled to −70°C. The backscattering configuration was used for the signal collection. The incident power on the samples was 7 mW. The spectra were recorded using a 40x objective, a 1 s accumulation time with a total of 10 accumulations. The synthesis of GSH-ZnS/Ag_2_Se core/shell QDs [16], GO nanosheets [17, 18] and CTAB-AuNPs [19] are described in the Electronic Supplementary Material.

### Conjugation of GO nanosheets to GSH-ZnS/Ag_2_Se core/shell QDs

**Scheme 1:**
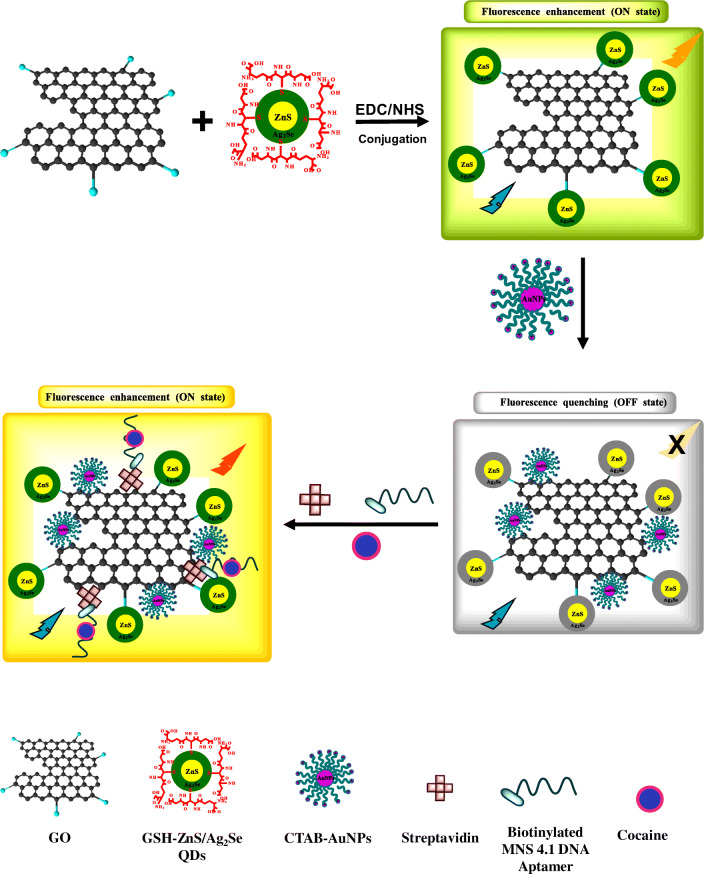
Descriptive representation of the strep-B-QDs-GO-CTAB-AuNP aptamer-based fluorescence nanoprobe for cocaine. GO is first conjugated to the QDs to form a QDs-GO nanocomposite which then results in fluorescence emission enhancement (ON state). Plasmonic CTAB-AuNP is then adsorbed to the QDs-GO nanocomposite to form a QDs-GO-CTAB-AuNP nanohybrid assembly and the fluorescence emission is quenched (OFF state). Streptavidin protein is then adsorbed onto the QDs-GO-CTAB-AuNP nanohybrid assembly and a biotinylated DNA anticocaine aptamer receptor is bonded to the adsorbed streptavidin. Cocaine being added to the system, binds to the aptamer receptor and this interaction switches on the fluorescence intensity of the strep-B-QDs-GO-CTAB-AuNP nanohybrid assembly in a concentration-dependent manner

Scheme 1 shows the process for the conjugation of GO to GSH-ZnS/Ag_2_Se core/shell QDs. To activate the carboxylic groups on GO, 20 mg GO was dissolved in 5 mL of MilliQ H_2_O and 10 mL 0.1 M EDC was added and stirred for ~10 min. Thereafter, 20 mg GSH-ZnS/Ag_2_Se QDs in 25 mL MilliQ H_2_O and 10 mL 0.1 M NHS were added and the solution was stirred for ~3 h. The QDs-GO fluorescent nanocomposite was purified by acetone via centrifugation and later dried.

### Preparation of strep-B-QDs-GO-CTAB-AuNP nanohybrid assembly

To form the QDs-GO-CTAB-AuNP nanohybrid assembly, 0.4 mg.mL^−1^ QDs-GO nanocomposite solution was mixed with a solution of CTAB-AuNPs (0.1 nM) in a 1:2 *v*/v ratio, yielding a final concentration of 0.06 nM QDs-GO-CTAB-AuNP. Thereafter, the QDs-GO-CTAB-AuNP solution (0.06 nM) was mixed with 0.5 mg.mL^−1^ streptavidin solution (in phosphate buffered saline (PBS), pH 7.4) in a 6:1 v/v ratio to aid adsorption of the protein onto the nanohybrid assembly. Subsequently, 10 μM biotinylated MNS 4.1 anticocaine DNA aptamer was added into the strep-QDs-GO-CTAB-AuNP solution and stirred to aid binding of the biotin end of the DNA aptamer to the streptavidin portion of the QDs-GO-CTAB-AuNP nanohybrid assembly. The formed strep-B-QDs-GO-CTAB-AuNP nanohybrid nanoprobe solution was stored at 4°C prior to use.

### Fluorescence bioassay

A novel buffer for cocaine determination was prepared by mixing 0.38 g MES, 0.25 g NaAc, 0.25 g KAc, 0.37 g KCl in 50 mL of MilliQ H_2_O and 6.7 mL 0.1 M HCl was added and made up to 100 mL with MilliQ H_2_O. Thereafter, the pH was adjusted to 2.2 using a solution of HCl and NaOH. For the fluorescence detection, appropriate cocaine concentration was dissolved in MES-NaAc-KAc-KCl-HCl buffer, pH 2.2 and 175 μl of the cocaine solution was mixed with 175 μl strep-B-QDs-GO-CTAB-AuNP nanohybrid nanoprobe solution. The fluorescence measurement was taken immediately in a quartz cuvette (~ 2 min) at an excitation wavelength of 290 nm within the photoluminescence (PL) wavelength range of 300 nm to 650 nm.

## Results and discussion

### Structural properties

**Fig. 1:**
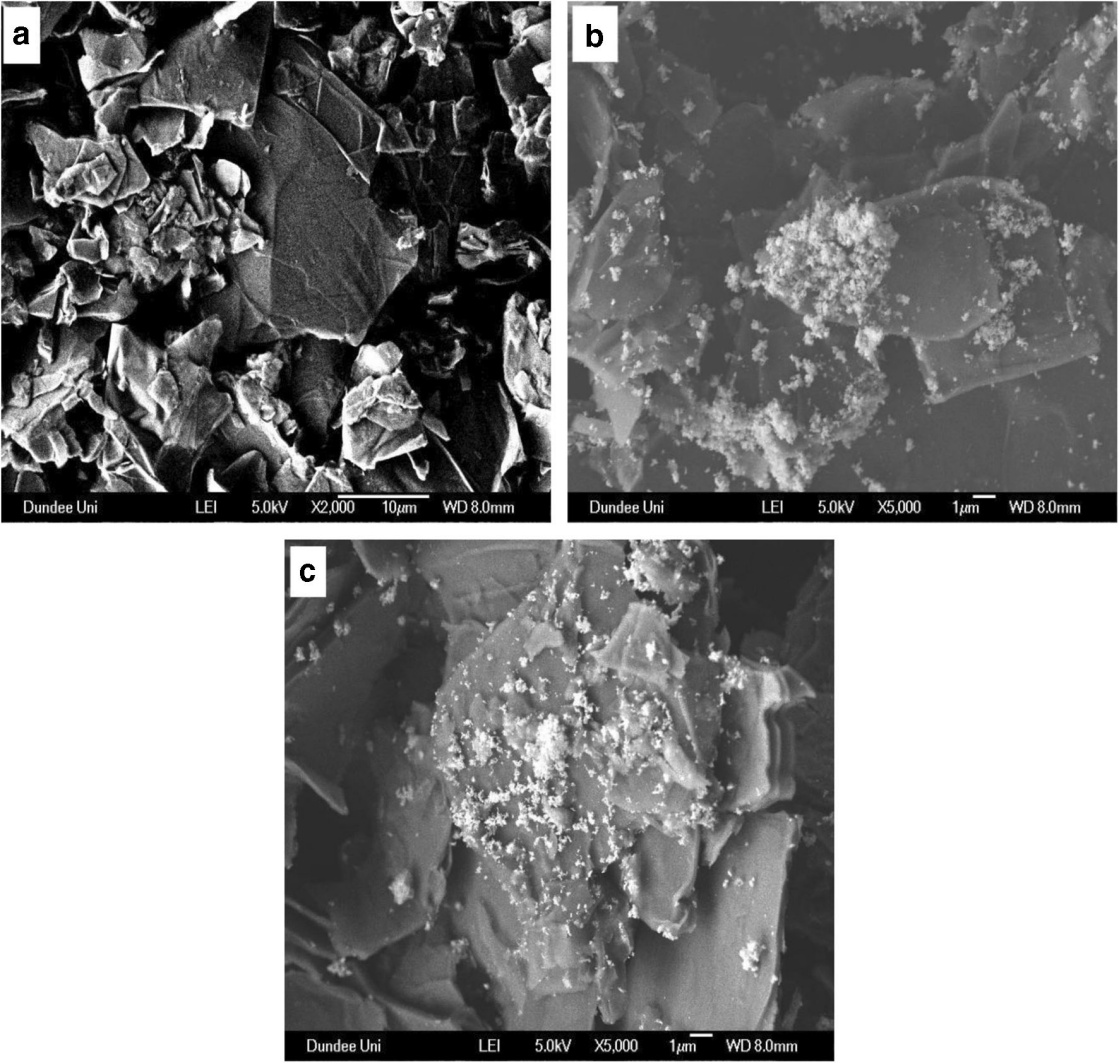
**a** SEM images of (**a**) GO, **b** QDs-GO nanocomposite and **c** QDs-GO-CTAB-AuNP nanohybrid

GO is known to be characterized by automatically thin and continuous two-dimensional (2D) carbon atom arrays that is embedded with carboxy groups on the edges and hydroxy and epoxy groups on the carbon basal plane [20]. Due to a significant portion of the basal plane being comprised of carbon domains, it is believed these regions of the graphene sheet are relatively hydrophobic [21]. However, GO is also thought to be composed of an amphiphilic structure which contributes to its aggregation and adsorption properties [21]. The SEM image of GO nanosheets obtained from chemical exfoliation of graphite oxide is shown in Fig. 1a. From the SEM image, GO nanosheets is observed to be characterized by crumpled and randomly aggregated thin sheets that folds like wrinkled silk waves into individual nanosheets. The corresponding SEM image of the QDs-GO nanocomposite (Fig. 1b) shows the presence of the QDs particles well anchored on the GO nanosheet surface. Similar features of embedded particles on the GO nanosheet surface was also observed for the QDs-GO-CTAB-AuNP nanohybrid assembly (Fig. 1c). From the observed morphological feature, it is noteworthy to affirm that both the QDs and plasmonic CTAB-AuNPs were well anchored on the GO nanosheet surface.

**Fig. 2:**
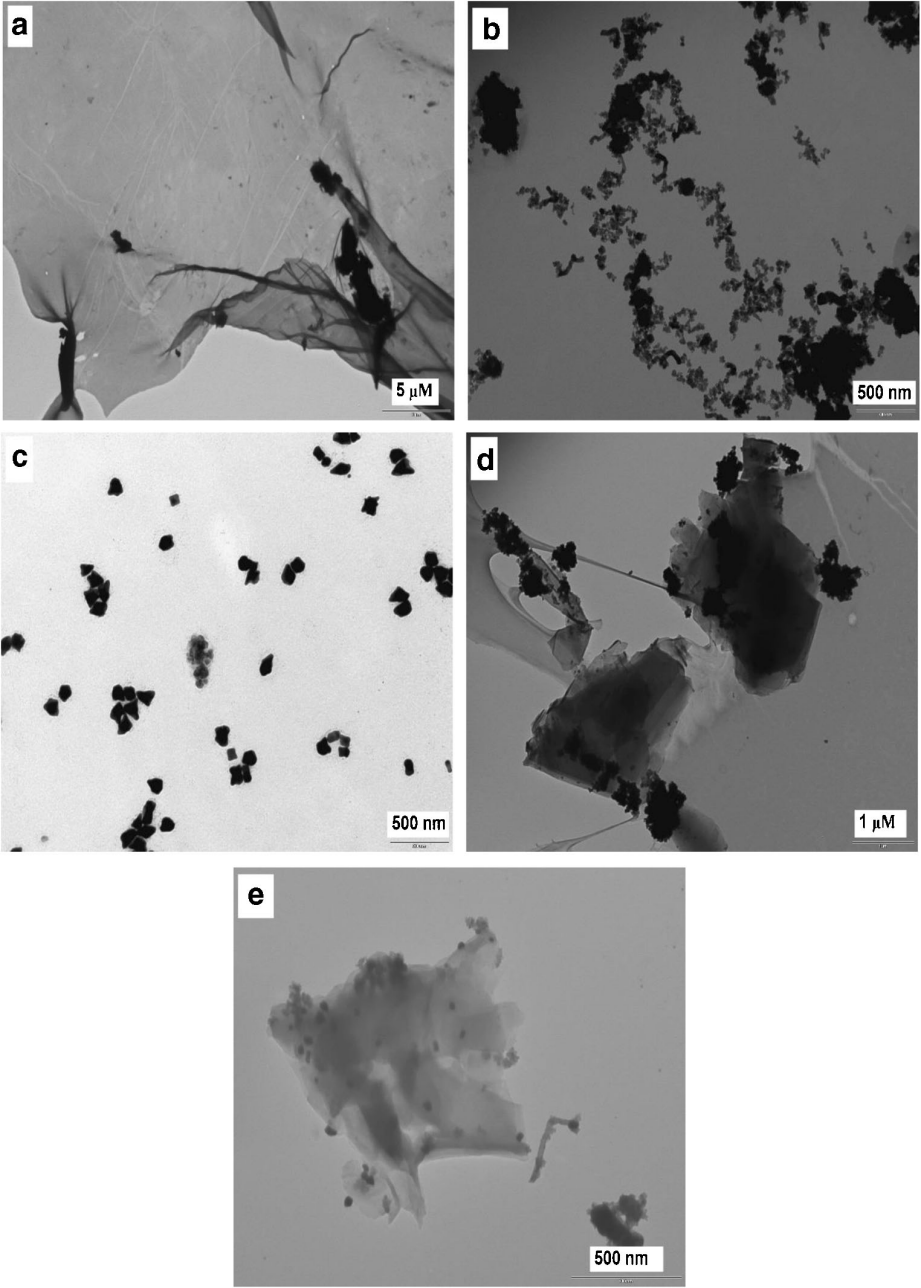
**a** TEM images of (**a**) GO, (**b**) GSH-ZnS/Ag_2_Se QDs, (**c**) CTAB-AuNPs, (**d**) QDs-GO nanocomposite and (**e**) QDs-GO-CTAB-AuNP nanohybrid

For the TEM analysis, Fig. 2a shows that the morphology of GO is characterized by exfoliated folded individual nanosheets with no trace of bulk aggregates. The TEM image of the newly synthesized GSH-ZnS/Ag_2_Se QDs was characterized by heterogenous QDs growth morphology as shown in Fig. 2b. Studies have shown that crystalline overcoating of a semiconductor layer may be formed on QDs nanocrystals either by ion displacement reactions or heterogeneous nucleation [22]. In some cases, it has been suggested that heterogeneous colloidal semiconductor particles exhibit better efficiency than single ensemble particles [23]. In general, shell coating on a core particle (in this case, the coating of Ag_2_Se shell on ZnS core) normally occurs via heterogenous nucleation. It is postulated that the direct deposition of the embryos of shell materials on the core surface, triggers the continuous formation of nuclei and QDs growth on the surface itself, instead of the formation of new nuclei in the bulk phase [23]. Therefore, the heterogenous formation of GSH-ZnS/Ag_2_Se QDs can be explained in terms of this phenomenon. Figure 2c shows the TEM image of CTAB-AuNPs. From the morphological display, the presence of cubic and prism-shaped particles was observed while most of the particles were irregularly-shaped. The average particle size was determined to be ~43 nm from 57 measured particles. Figure 2d and e shows the TEM images of the QDs-GO nanocomposite and the QDs-GO-CTAB-AuNP nanohybrid assembly. From the TEM images, it is apparent that GO sheets characterized by wrinkled surfaces were visibly seen for both images. Particularly, the binding of the QDs on the GO surface appears to be in lumps along the sheet edges. A close observation of the QDs-GO-CTAB-AuNP morphology reveal both the presence of particles spread across the sheet surface and the presence of lumps of particles along the sheet edge. Text and figures on the EDX analysis (Fig. S1), XRD pattern (Fig. S2), DLS and ZP analysis (Fig. S3) of the nanomaterials are provided in the Electronic Supporting Material.

### Optical properties

**Fig. 3:**
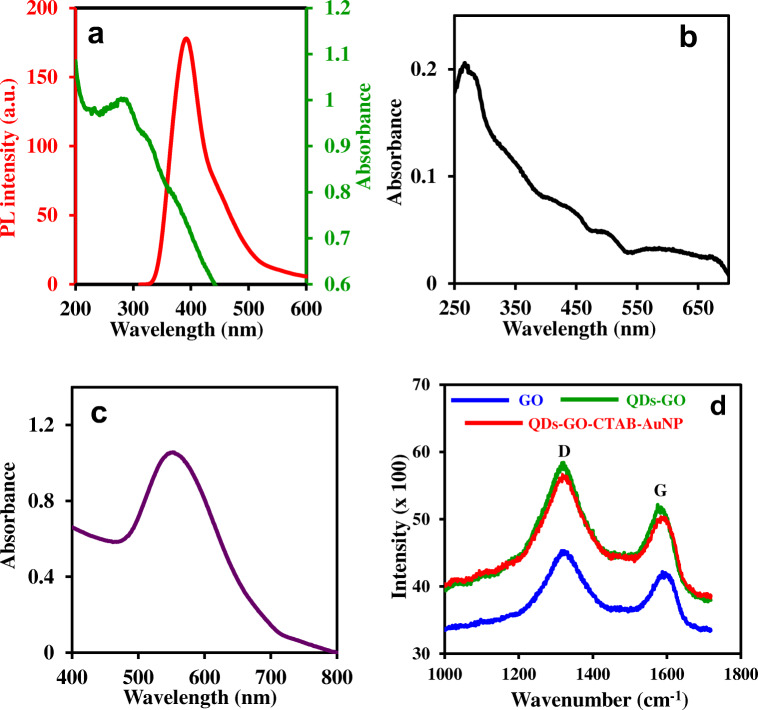
**a** UV/vis and PL emission spectra of GSH-ZnS/Ag_2_Se QDs. UV/vis absorption spectra of (**b**) GO and (**c**) CTAB-AuNPs. (**d**) Raman spectra of GO, QDs-GO nanocomposite and the QDs-GO-CTAB-AuNP nanohybrid. Λexc = 290 nm

Figure 3a shows the UV/vis absorption and PL emission spectra of the GSH-ZnS/Ag_2_Se QDs. The UV/vis absorption spectrum of the QDs is characterized by an absorption peak maximum at a wavelength of 288 nm and the PL emission spectrum is characterized by an emission wavelength at 395 nm. The UV/vis absorption spectrum of GO, shown in Fig. 3b is consistent with the absorption spectrum for the exfoliation of graphite oxide to GO and is distinct for a quasi-2D material. Consistent with published UV/vis spectrum for GO, the observed absorption maximum around 267 nm is attributed to the π → π* transition of the aromatic C-C bond [24], while the relatively broad absorption around 331–371 nm is attributed to the n → π* transition of the C = O bonds [25]. UV/vis absorption spectrum of the positively-charged plasmonic CTAB-AuNPs (Fig. 3c) is characterized by a distinct LSPR absorption band at 554 nm and is consistent with the LSPR absorption wavelength range for non-spherical AuNPs [26].

The characteristic D and G bands of carbon-based materials, projected by Raman analysis, have been used extensively to analyse the effects of carbon grain size, disorder and defect states on the carbon graphitic domain [27]. The Raman spectra of GO, QDs-GO nanocomposite and QDs-GO-CTAB-AuNP are shown in Fig. 3d. From the displayed spectra, GO is characterized by the D and G bands which is also visibly projected in the spectra of the QDs-GO nanocomposite and the QDs-GO-CTAB-AuNP nanohybrid. The D band corresponds to defects associated with amorphous carbon species, grain boundaries and vacancies, while the G band is associated with the E_2g_ mode of graphite which relates to sp^2^-bonded vibrational carbon atoms in a 2D hexagonal lattice [27]. From Fig. 3d, the D bands of GO, QDs-GO nanocomposite and QDs-GO-CTAB-AuNP are projected at 1336 cm^−1^, 1321 cm^−1^ and 1322 cm^−1^ while the G bands are projected at 1595 cm^−1^, 1575 cm^−1^ and 1584 cm^−1^ respectively. From the data, the binding of GO to the QDs and the subsequent adsorption of CTAB-AuNPs to the QD-GO nanocomposite induced both the D and G bands to shift to lower wavenumber relative to GO. The ratio of intensity (I_D_/I_G_) of the D band relative to the G band is usually used to determine the average size of the sp^2^ graphitic domain and the extent of defect/disorder degree [28]. The obtained I_D_/I_G_ value of 1.12 for the QD-GO nanocomposite and QDs-GO-CTAB-AuNP nanohybrid was higher than the value obtained for GO (I_D_/I_G_ = 1.06). Thus, it implies that the nanohybrids did not increase the ordered degree of graphitic carbon layers. Text and figures (Fig. S4A-C) on the FT-IR analysis of the QDs, GO, CTAB-AuNPs, QDs-GO nanocomposite and the QDs-GO-CTAB-AuNPs nanohybrid assembly are provided in the Electronic Supporting Material.

### Binding effects on fluorescence emission and LSPR absorption

**Fig. 4:**
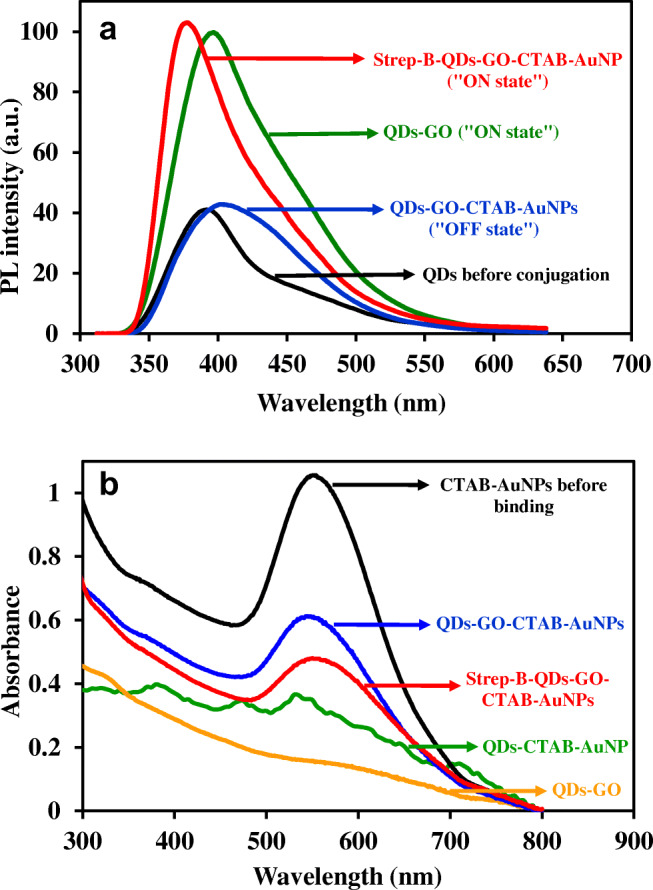
**a** PL emission spectra of the QDs before conjugation, QDs-GO nanocomposite, QDs-GO-CTAB-AuNP and strep-B-QDs-GO-CTAB-AuNP nanohybrid assembly. **b** UV/vis absorption spectra of CTAB-AuNPs before binding, QDs-GO nanocomposite, QDs-CTAB-AuNPs, QDs-GO-CTAB-AuNP and the strep-B-QDs-GO-CTAB-AuNP nanohybrid assembly

The effects of binding interactions on the fluorescence emission of the QDs and the LSPR absorption of the plasmonic CTAB-AuNPs was studied. Figure 4a shows the fluorescence emission changes induced by the binding of GO to the QDs, binding of CTAB-AuNPs to the QDs-GO nanocomposite and binding of the streptavidin-biotinylated DNA aptamer to the QDs-GO-CTAB-AuNP nanohybrid. From the data, the binding of GO to the QDs resulted in marked fluorescence enhancement (ON state) and a red-shift in emission wavelength (17 nm) relative to the fluorescence of the unconjugated QDs. The red-shift may be attributed to agglomeration of the QDs upon binding to GO. Upon adsorption of the plasmonic CTAB-AuNPs to the QDs-GO nanocomposite, the fluorescence was quenched, leading to the fluorescence of the conjugated QDs being switched off. The subsequent binding interaction between streptavidin and the biotinylated DNA aptamer on the QDs-GO-CTAB-AuNP nanohybrid assembly, led to the fluorescence being switched on and a blue- shift of the emission wavelength. The blue shift in emission wavelength can be attributed to disaggregation of the QDs upon binding of the protein and DNA aptamer. Thus, the strep-B-QDs-GO-CTAB-AuNP nanoprobe can function as an “ON-OFF-ON” aptamer-based fluorescence nanoprobe for cocaine.

Figure 4b shows the comparative binding changes on the LSPR absorption feature of CTAB-AuNPs. From the data, the binding of the QDs and GO individually to CTAB-AuNPs led to disappearance of the LSPR absorption. However, when CTAB-AuNPs was directly bonded to the QDs-GO nanocomposite, the LSPR absorption peak was well projected but relatively quenched in comparison to CTAB-AuNPs. The subsequent binding of the streptavidin-biotinylated DNA aptamer to the QDs-GO-CTAB-AuNP nanohybrid did not induce disappearance of the LSPR absorption peak but rather led to further reduction of the LSPR absorption. Based on the observed result, it is noteworthy to suggest that the presence of GO in the nanohybrid system, created a platform for retaining the LSPR absorption characteristic of the plasmonic CTAB-AuNPs, which is essential as a signal amplifier for ultrasensitive fluorescence determination of the target cocaine.

### Working principle of the nanoprobe (Scheme 1)

GO was first conjugated to the QDs to form the QDs-GO nanocomposite via EDC/NHS carbodiimide conjugation chemistry involving the formation of an amide bond linkage between the carboxylic groups on GO and the amino group on the QDs. The binding interaction triggered a fluorescence enhancement process, which is a direct contrast to the quenching effects of GO on QDs fluorescence as reported in the literature [29–31]. It is also noteworthy to emphasize that the fluorescence enhancement effect of GO on QDs [17] and on a molecular fluorophore [32] have been reported in the literature. Förster resonance energy transfer (FRET) from the donor QDs to the acceptor GO has been the most popular mode of quenching interaction reported between fluorescent-emitting QDs and GO [33]. Even though we observed a spectral overlap between the QDs fluorescence and GO absorption spectrum as shown in Fig. S5A, the absence of fluorescence quenching indicates that a different mode of chemical interaction occurred between the QDs and GO. Based on reported interpretation on the fluorescence enhancement effect of GO on QDs fluorescence [32], we believe that when the band gap of the QDs is less than or equal to the phonon energies, photoexcited electrons from the valence to the conduction band can induce equal amounts of holes in the valence band [34]. Judging by the morphology of the QDs shown via the TEM image, it is likely possible that interfacial surface defect created non-radiative electron-hole dynamics on the QDs surface. Therefore, we believe that conjugation of GO to the QDs induced conformability between the defect state of the QDs and the adsorptive properties of GO which may have induced fluorescence enhancement of the QDs [32].

**Fig. 5:**
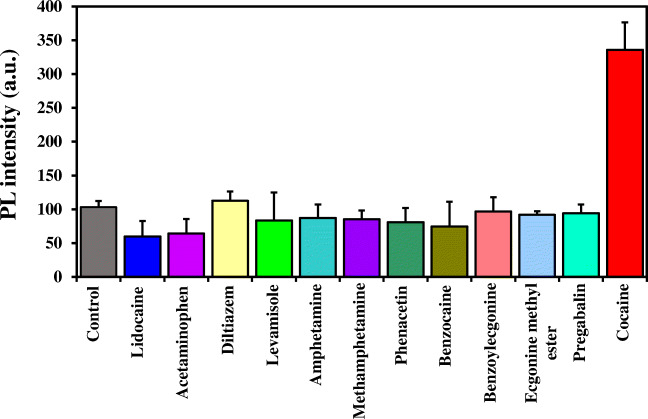
Selectivity of the strep-B-QD-GO-CTAB-AuNP aptamer nanoprobe to cocaine in comparison to other drugs and cocaine metabolites. Concentration of cocaine and other tested drugs and substances = 100 μM. Control = strep-B-QDs-GO-CTAB-AuNP solution without cocaine. Error bars are standard deviation of 3 replicate analysis

CTAB-AuNPs were subsequently bound to the QD-GO nanocomposite and can bind to the conjugated-GO via electrostatic interaction between the CTAB headgroup and free OH groups on GO and via hydrophobic interaction involving the hydrophobic alkyl chains of CTAB [35]. The binding of CTAB-AuNPs then triggered fluorescence quenching of the QDs-GO-CTAB-AuNP nanohybrid assembly. To investigate the fluorescence quenching effect of CTAB on the QDs-GO nanocomposite, we have displayed the spectral overlap between the UV/vis absorption spectrum of CTAB and the fluorescence emission spectrum of the QDs-GO nanocomposite in Fig. S5B. The displayed spectral overlap suggests that FRET must have led to the fluorescence quenching process. However, from Fig. 5b, the absorption spectrum of the QD-GO-CTAB-AuNPs was quenched relative to the unbonded CTAB-AuNPs, suggesting that FRET was not the mechanism for the quenching process between the QDs-GO nanocomposite and CTAB-AuNPs. Therefore, we believe that the direct suppression of the radiative decay rate of the QDs-GO emission by CTAB-AuNPs must have led to the quenching process [36]. To aid binding of the biotinylated DNA aptamer, streptavidin was first adsorbed on the QDs-GO-CTAB-AuNP nanohybrid resulting in a streptavidin-biotin DNA-aptamer interaction on the surface of the nanohybrid. The incorporation of cocaine into the strep-B-QDs-GO-CTAB-AuNP system, induced marked fluorescence enhancement process in a concentration dependent manner. The MNS-4.1 anticocaine aptamer is known to have a strong affinity for cocaine. A target-induced conformation change in the form of a noncanonical three-way junction is formed upon binding of the unfolded aptamer with cocaine (Fig. S6) [1]. This binding interaction then induced fluorescence transduction changes in the nanoprobe, resulting in a fluorescence switched on process for cocaine determination.

### Optimization of method

The following parameters were optimized: (a) Sample pH, (b) nanoprobe efficiency and (c) LSPR effect from different plasmonic NPs. Respective text and figures on optimization (Fig. S7A-C) are given in the Electronic Supporting Material. In general, the following experimental conditions were found to give best results: (a) Optimal pH value = pH 2.2, (b) optimal probe efficiency = strep-B-QDs-GO-CTAB-AuNP and (c) optimal LSPR effect = CTAB-AuNPs.

### Selectivity of the nanoprobe

The selectivity of the strep-B-QDs-GO-CTAB-AuNP aptamer-based fluorescence nanoprobe for cocaine determination was studied in the presence of other drugs and substances and cocaine metabolites. Figure 5 shows the selectivity data for cocaine determination using the aptamer-based fluorescence nanoprobe. From the data, a significant and superior fluorescence intensity signal was obtained for cocaine (100 μM) in comparison to the other tested drugs and substances (100 μM). Particularly, the nanoprobe was insensitive to the tested cocaine metabolites (benzoylecgonine and ecgonine methyl ester (100 μM)), thus affirming the unique selectivity of the nanoprobe to cocaine alone. Based on the obtained results, we can say with confidence that the strep-B-QDs-GO-CTAB-AuNP aptamer-based fluorescence nanoprobe can successfully be used for the selective determination of cocaine and confirm its presence in a sample.

### Quantitative cocaine determination

**Fig. 6:**
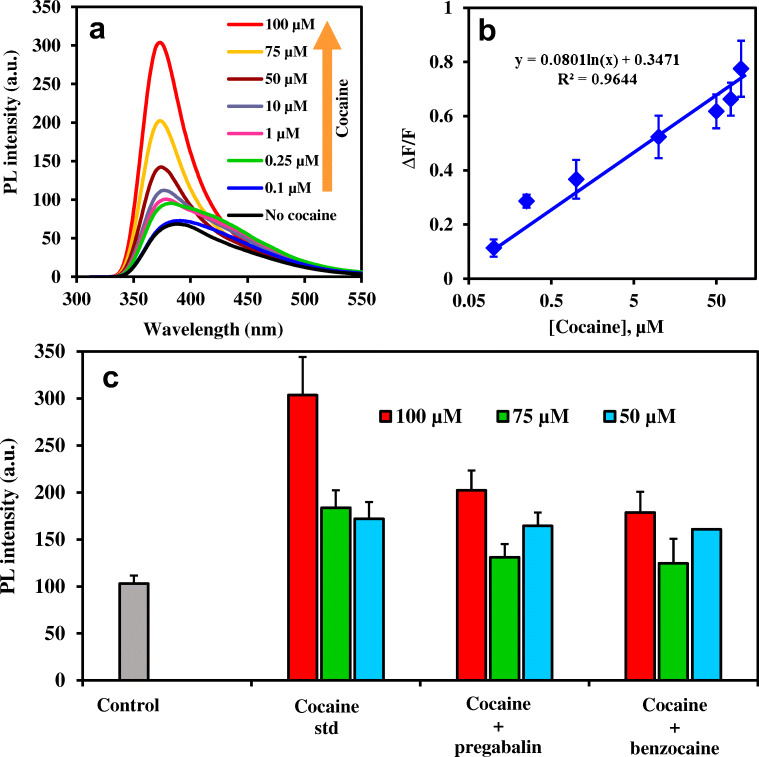
**a** Fluorescence “turn ON” spectra of the strep-B-QDs-GO-CTAB-AuNP aptamer nanoprobe to cocaine quantitative determination, **b** Photoluminescence (PL) intensity calibration plot for cocaine quantitative determination, **c** PL intensity signal for the determination of 100, 75 and 50 μM pure cocaine standard in comparison to adulterated cocaine samples containing benzocaine and pregabalin. Error bars are standard deviation of 3 replicate analysis. Λexc = 290 nm. Maximum PL intensity signal taken at ~370 nm

Quantitative determination of cocaine was carried in the concentration range of 0.1–100 μM by the strep-B-QDs-GO-CTAB-AuNP aptamer-based fluorescence nanoprobe. Figure 6a shows the fluorescence emission spectral changes for each concentration of cocaine detected. From the data, the fluorescence of the aptamer-based strep-B-QDs-GO-CTAB-AuNP nanoprobe was switched on in a concentration-dependent manner. A slight red shift and broadening of the fluorescence emission spectrum was observed at low concentrations of cocaine. This can be attributed to the aptamer folding interaction with low concentration of cocaine and its subsequent effect on the optical property of the fluorophore nanoprobe. The corresponding fluorescence linear plot, shown in Fig. 6b was used to determine the limit of detection (LOD) of the nanoprobe by multiplying the standard deviation of blank measurement (*n* = 9) by 3 and dividing by the slope of the linear plot. The LOD obtained for cocaine determination using the strep-B-QDs-GO-CTAB-AuNP aptamer-based fluorescence nanoprobe was 4.6 nM (1.56 ng.mL^−1^) while the linear range was from 0.1–100 μM. Comparison of the analytical performance of the developed nanoprobe with other published probes for cocaine (Table 1) shows that our detection system exhibited a more favourable balance between sensitivity and response time than most probes.

**Table 1:** Comparison of the analytical performance of the strep-B-QDs-GO-CTAB-AuNP aptamer-based fluorescence nanoprobe with other published aptamer-based fluorescence nanoprobe for cocaine

Strategy	Method	LOD	Detection time	Ref.
FRET aptamer assay	Fluorescence turn-OFF	10 μM	–	[37]
Structure-switching aptamers-SYBR Gold-exonuclease I	Fluorescence turn-ON	5 μM	25 min	[7]
2-amino-5,6,7-trimethyl-1,8-naphthyridine aptamer assay	Fluorescence turn-ON	200 nM	20 s	[1]
QDs-FRET aptamer assay	Fluorescence turn-OFF and turn-ON	0.5 μM	1 min 40 s	[8]
Strand displacement amplification aptamer assay	Fluorescence turn-ON	1 nM	2 h	[38]
G-Quadruplex-Iridium (III) complex aptamer assay	Fluorescence turn-ON	30 nM	30 min	[4]
2-Amino-5,6,7-trimethyl-1,8- naphthyridine-SYBR Green I aptamer assay	Fluorescence turn-ON	56 nM	20 s	[39]
GO and exonuclease III-assisted signal amplification	Fluorescence turn-ON	0.1 nM	17 min	[9]
Strep-B-QDs-GO-CTAB-AuNP aptamer assay	Fluorescence turn-ON	4.6 nM	~ 2 min	This work

### Determination of cocaine in seized adulterated samples

The applicability of the strep-B-QDs-GO-CTAB-AuNP aptamer-based fluorescence nanoprobe to detect seized adulterated cocaine samples was investigated. Seized cocaine samples containing benzocaine and pregabalin adulterants were detected at 100, 75 and 50 μM and the obtained fluorescent signals were compared to the signal obtained for a pure cocaine standard sample. Figure 6c shows that the strep-B-QDs-GO-CTAB-AuNP aptamer-based fluorescence nanoprobe was efficient in detecting adulterated cocaine within the sample. As expected, the fluorescent intensity was lower in comparison to the pure cocaine sample due to the adulterated nature of the drug sample. Thus, it is imperative to conclude that the constructed strep-B-QDs-GO-CTAB-AuNP aptamer-based fluorescence nanoprobe is suitable for the real-life determination of both pure and adulterated cocaine samples.

An attempt to detect cocaine using the strep-B-QDs-GO-CTAB-AuNP aptamer-based fluorescence nanoprobe in spiked urine sample was unsuccessful. Despite this limitation, most illicit cocaine samples are illegally produced and transported in white or off-white crystalline powder and their physical appearance is only slightly changed in adulterated samples which themselves are known to appear in fine dry white powders. As we have demonstrated in our work, our nanoprobe is sensitive and selective enough to detect cocaine and its adulterated sample in their crystalline powdered form.

## Conclusions

A novel strep-B-QDs-GO-CTAB-AuNP aptamer-based fluorescence nanoprobe platform has been developed for cocaine. Under optimum experimental conditions, the constructed QDs-GO-CTAB-AuNP nanohybrid was found to exhibit far superior sensitivity to cocaine than the tested strep-B-QDs (no GO and CTAB-AuNPs), strep-B-QDs-CTAB-AuNP (no GO) and strep-B-QDs-GO (no CTAB-AuNP). Also, LSPR signal form CTAB-AuNPs was found to amplify the fluorescence intensity in superior fashion than other tested plasmonic NPs. In general, the new aptamer-based fluorescent nanoprobe platform exhibited low detection limit, excellent selectivity and rapid response time. The application of the nanoprobe to detect seized adulterated cocaine was successfully achieved.

## Electronic supplementary material

ESM 1 (DOCX 1663 kb)
