# Supplementary material for: Aptamer-based cocaine assay using a nanohybrid composed of ZnS/Ag2Se quantum dots, graphene oxide and gold nanoparticles as a fluorescent probe
Source: Mikrochim Acta. 2020 Jan 8;187(2):104. doi: 10.1007/s00604-019-4101-6 (PMC6946730; doi:10.1007/s00604-019-4101-6)
Supplement: Supplementary file 1 — (DOCX 1663 kb) [file 604_2019_4101_MOESM1_ESM.docx]

**Electronic Supporting Material**

**Aptamer-based assay for cocaine using a nanohybrid composed of ZnS/Ag_2_Se quantum dots, graphene oxide and gold nanoparticles as a fluorescent probe**

Oluwasesan Adegoke^1^*, Magda A. Pereira-Barros^1^, Svetlana Zolotovskaya^2^, Amin Abdolvand^2^, Niamh Nic Daeid^1^

*^a^Leverhulme Research Centre for Forensic Science, University of Dundee, Dundee, DD1 4GH, UK*

*^b^Materials Science & Engineering Research Cluster, School of Science & Engineering, University of Dundee, DD1 4HN, UK*

**Synthesis of GSH-****ZnS/Ag_2_Se core/shell QDs**

The synthesis of ZnS/Ag_2_Se QDs was carried out according to the traditional organometallic synthetic hot-injection pyrolysis of QDs. In a three-necked flask fitted with a refluxing condenser, 5 mL of diethylzinc was mixed with 1.8 g trioctylyphosphine oxide, 2 mL trioctylyphosphine, 0.6 g hexadecylamine, 0.6 g myristic acid, 20 mL octadecene, 2 mL oleylamine and 15 mL oleic acid. The solution was then bubbled with N_2_ gas and heated to ~320°C to allow complexation of the Zn to the precursors. Thereafter, the sulphur solution containing a mixture of 0.16 g sulphur, 0.9 g trioctylyphosphine oxide, 1 mL trioctylyphosphine and 10 mL octadecene was added into the hot Zn-complexed solution to allow the nucleation and growth of the ZnS core. To overcoat the Ag_2_Se shell layer, 1 mL of Ag precursor, prepared by dissolving 0.5 g AgNO_3_ in 5 mL trioctylyphosphine and heating until the Ag salt was completely dissolved, was added into the hot ZnS solution. This was followed swiftly by the addition of 3 mL Se solution, prepared by dissolving 0.5 g Se in 5 mL trioctylyphosphine. The organic-phased ZnS/Ag_2_Se QDs was left to nucleate and grow for ~15 min and thereafter harvested and dispersed in chloroform.

Ligand exchange reaction to convert the hydrophobic QDs to hydrophilic nanocrystals was carried out by mixing 3 g of KOH and 5 g GSH in 40 mL methanol. The solution was then sonicated to aid the complete dissolution of KOH and GSH. Thereafter, the chloroform-dispersed ZnS/Ag_2_Se QDs was added into the GSH-KOH-methanolic solution, stirred and allowed to stand still for ~24 hours (hr). The GSH-ZnS/Ag_2_Se core/shell QDs were purified using acetone (step 1), acetone/ethanol/chloroform (step 2), H_2_O/chloroform/acetone (step 3), ethanol/acetone (step 4) and acetone (step 5).

**Synthesis of GO nanosheets**

GO nanosheets were synthesized according to the modified Hummer’s method. Briefly, 60 mL of H_2_SO_4_ in a round bottom flask was first cooled with ice. Then 1.25 g of NaNO_3_ and 2.5 g graphite powder were added and stirred vigorously to aid thorough dispersion of the graphite powder. Subsequently, 7.5 g of KMnO_4_ was added into the graphite solution, stirred and the solution kept in ice for ~2 hours. Thereafter, the reaction mixture was stirred for ~24 hours at room temperature and subsequently placed in an ice bath and this was followed by the slow addition of 75 mL of MilliQ water. Thereafter, the reaction mixture was stirred at 100°C for ~24 hr and allowed to cool down before the slow addition of 25 mL of 35% H_2_O_2_. The prepared graphite oxide paste was then centrifuged with 5% HCl followed by acetone to remove unreacted metal ion and subsequently dried at 65°C. GO nanosheets were obtained by exfoliation of graphite oxide via ultrasonication for ~3 hr.

**Synthesis of CTAB-AuNPs**

CTAB-AuNPs were prepared according to the seed-mediated approach as described in the literature. Seed solution containing 10 mL 0.1 M CTAB, 5 mL 2.5 × 10^-4^ M HAuCl_4_.3H_2_O and 0.6 mL NaBH_4_ was first prepared. Thereafter, 12 µl of the seed solution was added into a growth solution containing 10 mL 0.1 M CTAB, 0.4 mL 0.1 M ascorbic acid, 4 mM AgNO_3_ and 5 mL 2.5 × 10^-4^ M HAuCl_4_.3H_2_O. The reaction was stirred and allowed to stand still for ~24 hr. Purification of the plasmonic NPs were carried via centrifugation and thereafter re-suspended in MilliQ H_2_O.

**Structural properties**

EDX was used to qualitatively and quantitatively analyse the elemental composition of the QDs, QDs-GO nanocomposite and the QDs-GO-CTAB-AuNP nanohybrid. Fig. S1A shows that the EDX spectrum of the QDs consist of the metal components of Zn, S, Ag, Se, C and O and these metals were also detected in the EDX spectra of the QDs-GO nanocomposite (Fig. S1B) and the QDs-GO-CTAB-AuNP nanohybrid (Fig S1C). The Au metal component from the plasmonic CTAB-AuNPs was additionally detected in the EDX spectrum of the QDs-GO-CTAB-AuNP nanohybrid. Assessing the quantitative elemental composition (Table S1), we observed that the QDs were rich in Zn. An important observation was the increase in C and O composition in the QDs-GO relative to the unconjugated QDs and further increase as CTAB-AuNPs were bonded to the QDs-GO nanocomposite. The increase in C and O composition after binding can be attributed to the presence of the graphitic carbon domain and oxygen functional groups of GO. The carbon from the surfactant CTAB could also contribute to the increase in C composition for the QDs-GO-CTAB-AuNP nanohybrid.


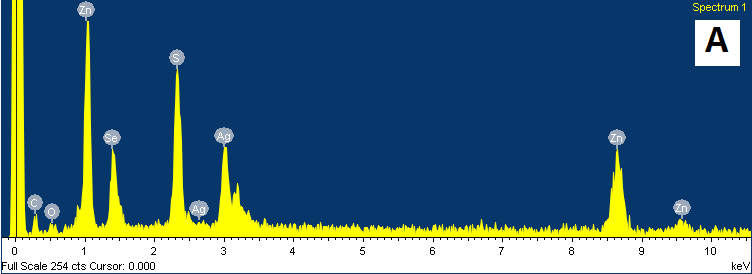


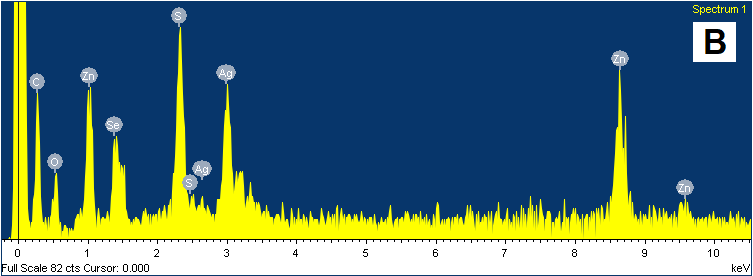


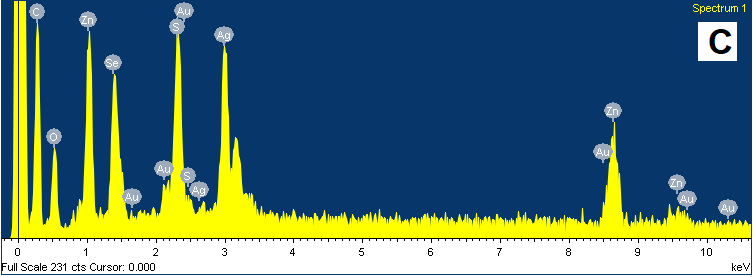


**Fig. S1.** EDX spectra of (A) GSH-ZnS/Ag_2_Se QDs and (B) QDs-GO nanocomposite and (C) QDs-GO-CTAB-AuNPs nanohybrid assembly.

The crystal structural phase of GO, GSH-ZnS/Ag_2_Se QDs, CTAB-AuNPs and the nanohybrids was studied using PXRD. From Fig. S2A, the well-pronounced diffraction peak for GO at ~20° corresponds to the {002} plane of the crystalline hexagonal structure of GO **[1]**. The broad diffraction peak is an indication of embedded multi-layered domain of GO sheets. The diffraction pattern of GSH-ZnS/Ag_2_Se QDs corresponds significantly to a cubic diffraction domain with orthorhombic Ag_2_Se structure and fits well to the Naumannite JCPDS card no. 71-2410 **[2]**. Examining the diffraction pattern of the QDs-GO nanocomposite, it is evident that the diffraction pattern resembles a combination of the QDs and GO. Thus, it is imperative to suggest that the covalent conjugation of the QDs to GO is reflected in the diffraction pattern of the QD-GO nanocomposite. Probing the diffraction pattern of the QDs-GO-CTAB-AuNP nanohybrid assembly (Fig. S2B) after binding CTAB-AuNPs to the QDs-GO nanocomposite, it is evident that the diffraction pattern is dominated by the {002} diffraction peak in GO with less visible trace of the diffraction peaks of CTAB-AuNPs and the QDs.

**Fig S2.** (A) XRD pattern for the GO, QDs, and QDs-GO nanocomposite and (B) CTAB-AuNPs and the QDs-GO-CTAB-AuNP nanohybrid assembly.

**DLS and ZP analysis**

DLS also known as quasi-elastic light scattering or photon correlation spectroscopy is popularly used to determine the hydrodynamic size of particles. DLS measures as a function of time, the hydrodynamic radius (R_H_) of the dispersed particles based on the analysis of the projected scattered light intensity that penetrates through the colloidal solution. In general, smaller particles diffuse faster than larger particles and based on the time-dependent scattered light, a mathematical function is produced that correlates to the measured particle size. DLS can therefore be used to assess the aggregation state of a colloidal solution. ZP on the other hand, was used to probe the surface charge of the GO, QDs, CTAB-AuNPs and the strep-B-QDs-GO-CTAB-AuNPs nanoprobe. Fig. S3 shows the DLS hydrodynamic size curves and ZP curves for GO, QDs, CTAB-AuNPs and the strep-B-QDs-GO-CTAB-AuNP nanohybrid while Table S2 list the corresponding values.


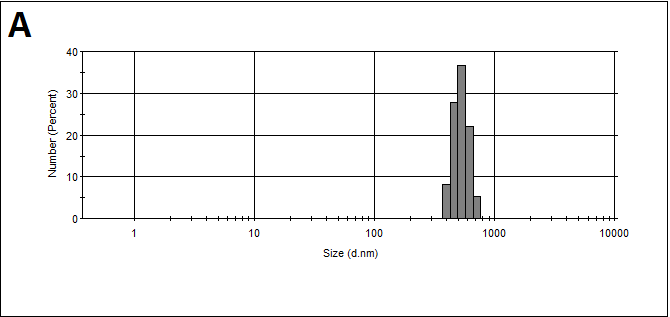


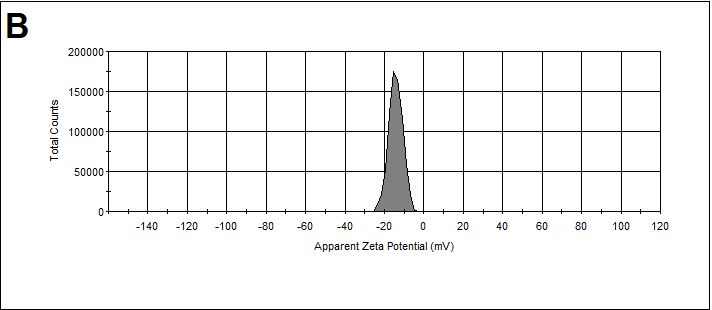


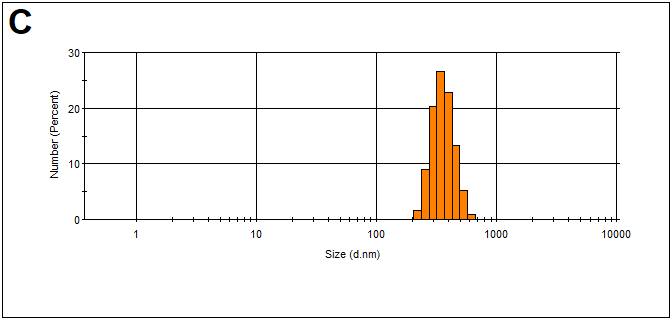


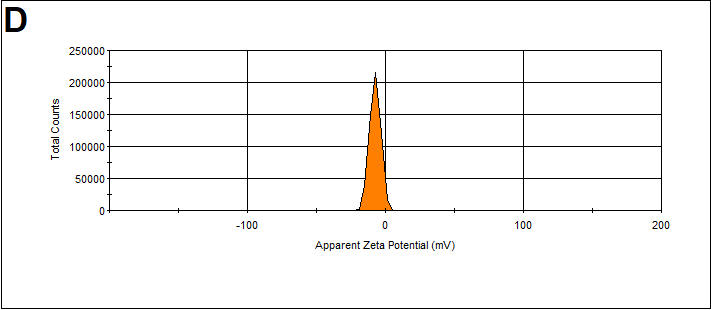


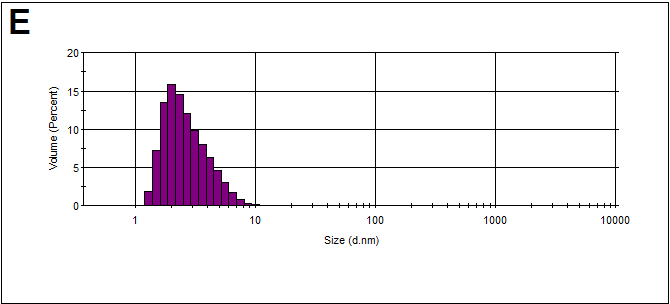


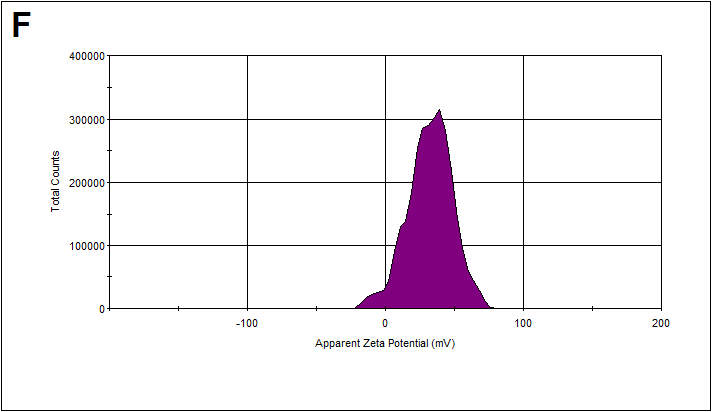


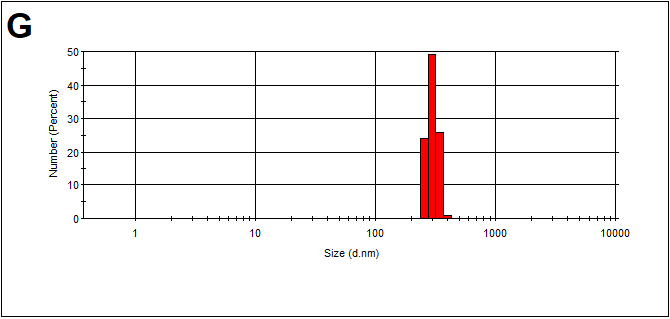


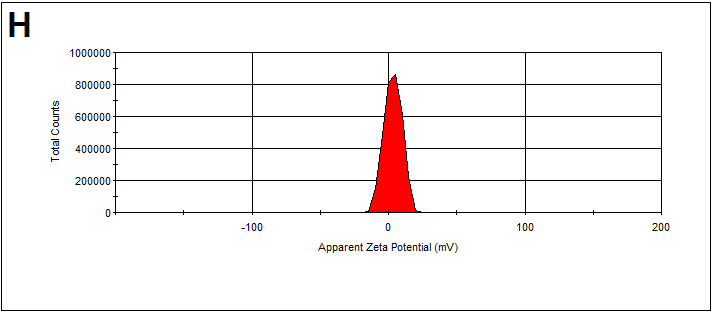


**Fig. S3.** DLS hydrodynamic size curves and Zeta potential curves for GO (A, B), GSH-ZnS/Ag_2_Se QDs (C, D), CTAB-AuNPs (E, F) and the strep-B-QDs-GO-CTAB-AuNP nanohybrid (G, H).

**Optical Properties**

FT-IR analysis was used to probe the functional groups of GO, GSH-ZnS/Ag_2_Se QDs, CTAB-AuNPs and the nanohybrids. From Fig. S4A, GO is characterized by a broad O-H stretch which is derived from the COOH group (υ_O-H_ = 3351 cm^-1^), carboxyl/carbonyl (υ_C=O_ = 1701 cm^-1^), aromatic (υ_C=C_ = 1560 cm^-1^), carboxy stretching group (υ_C-O_ = 1363 cm^-1^), epoxy (υ_C-O_ = 1221 cm^-1^) and alkoxy functional group (υ_C-O_ = 1043 cm^-1^). The QDs is characterized by the presence of symmetric (υ_C=O_ = 1398 cm^-1^) and asymmetric (υ_C=O_ = 1560 cm^-1^) carboxyl/carbonyl functional groups and a broad OH stretch group (υ_C=O_ = 3282 cm^-1^). From close examination of the FT-IR spectrum of the QDs-GO nanocomposite, it is evident that the spectrum is dominated by GO. However, the band at 1717 cm^-1^ is slightly shifted to higher wavenumber relative to GO and one could attribute the shift to the formation of amide linkage between GO and the QDs **[3]**. The FT-IR spectrum of CTAB-AuNPs (Fig. S4B) is characterized by the -C-H stretch (υ_C-H_ = 2846 cm^-1^ and υ_C-H_ = 2913 cm^-1^) and -C-H bend (υ_C-H_ = 1478 cm^-1^). For the QDs-GO-CTAB-AuNP nanohybrid (Fig. S4C), the band at 2361 cm^-1^ which was not present in the FT-IR spectra of GO, the QDs and QDs-GO nanocomposite, can be attributed to the C=N stretching vibration for a nonconjugated system. Since CTAB-AuNPs was adsorbed onto the QDs-GO nanocomposite surface via a nonconjugated approach, the appearance of the band at 2361 cm^-1^ may confirm the adsorption binding process.

**Fig. S4.** FT-IR spectra of (A) GSH-ZnS/Ag_2_Se QDs, GO and the QDs-GO nanocomposite; (B) CTAB-AuNPs and (C) QDs-GO-CTAB-AuNPs nanohybrid assembly.

**Fig. S5.** Spectral overlap between the UV/vis absorption spectrum of GO and the fluorescence emission spectrum of the QDs (A) and spectral overlap between the UV/vis absorption of CTAB-AuNPs and the fluorescence emission spectrum of the QDs-GO nanocomposite.

**Fig. S6.** Typical noncanonical three-way junction formed upon binding of the MNS-4.1 DNA aptamer to cocaine.

**Effects of pH**

The effects of pH on the detection of cocaine using the strep-B-QDs-GO-CTAB-AuNP aptamer-based fluorescence biosensor was studied. As shown in Fig. S7A, the pH of the buffered cocaine solution was studied in the pH range of 2.2. to 5.0. At a fixed concentration of cocaine detected (100 µM), the fluorescence intensity of the strep-B-QDs-GO-CTAB-AuNP aptamer-based fluorescence biosensor was superiorly enhanced at pH 2.2 in comparison to the rest of the tested pH. As the pH increased, the fluorescence intensity signal decreased down to pH 4.2 and then increased slightly to pH 5.0. Based on the observed result, pH 2.2 was chosen as the choice pH for the bioassay. It noteworthy to point out that the stability of cocaine at pH 2.2 **[4,5]** and the hydrolysis of cocaine to its metabolites (benzoylecgonine and ecgonine methyl ester) at high pH have been reported in literature **[6].**

**Probe comparison**

The efficiency of the strep-B-QDs-GO-CTAB-AuNP biosensor to detect cocaine with superior sensitivity was studied in comparison to strep-B-QDs (no GO and CTAB-AuNPs), strep-B-QDs-CTAB-AuNP (no GO) and strep-B-QDs-GO (no CTAB-AuNP). Fig. S7B shows the sensitivity data with respect to the fluorescence intensity enhancement of the tested probes. In the presence of 100 µM cocaine concentration, each of the tested probe induced varying degree of the fluorescence signal enhancement that was equivalent to its sensitivity. Without the presence of GO and CTAB-AuNPs in the biosensor system (strep-B-QDs), the fluorescence signal of the detected cocaine was very low. Also, very low fluorescence signal intensity was observed for the probe when GO was absent from the system (strep-B-QDs-CTAB-AuNP). When CTAB-AuNPs was absent from the biosensor system (strep-B-QDs-GO), the fluorescence signal was moderately enhanced. However, for the strep-B-QDs-CTAB-AuNP aptamer fluorescent probe, the fluorescence intensity was superiorly enhanced in comparison to the other tested probes. Therefore, the constructed strep-B-QDs-GO-CTAB-AuNP nanohybrid aptamer fluorescent probe can function as an ultrasensitive fluorescent probe for cocaine.

**LSPR effect**

The effect of LSPR from different plasmonic NPs to amplify the fluorescence signal intensity of the strep-B-QDs-GO-CTAB-AuNP aptamer-based fluorescent biosensor for cocaine detection was studied. Citrate-AuNPs, citrate-AgNPs and citrate-AuAgNPs were each adsorbed to the QDs-GO nanocomposite and bonded to the streptavidin-biotin DNA aptamer and tested as fluorescent probes in comparison to the strep-B-QDs-GO-CTAB-AuNP biosensor probe. Fig. S7C shows that each of the tested plasmonic citrate-AuNPs, citrate-AgNPs and citrate-AuAgNPs quenched the fluorescence signal but CTAB-AuNPs amplified the signal for cocaine detection. Thus, the strep-B-QDs-GO-CTAB-AuNP nanohybrid can function as an aptamer-based LSPR-amplified fluorescence intensity biosensor for cocaine.

**Fig. S7.** (A) Effects of pH on the fluorescence detection of cocaine using the strep-B-QDs-GO-CTAB-AuNP aptamer biosensor. (B) Enhanced PL sensitivity of the strep-B-QDs-GO-CTAB-AuNP aptamer biosensor in comparison to the strep-B-QDs alone (without GO and CTAB-AuNPs), strep-B-QDs-GO (without CTAB-AuNPs) and strep-B-QDs-CTAB-AuNPs (without GO). (C) Enhanced PL sensitivity of the strep-B-QDs-GO-CTAB-AuNP aptamer biosensor with incorporated CTAB-AuNPs in comparison to other embedded plasmonic NPs. Other plasmonic NPs bonded to the QDs-GO nanocomposite are citrate-AuNPs, citrate-AgNPs and bimetallic citrate-AuAgNPs. [Cocaine] = 100 µM; Control = strep-B-QDs-GO-CTAB-AuNP solution without cocaine. Error bars are standard deviation of 3 replicate analysis.

**Table S1.** Quantitative EDX data for GSH-ZnS/Ag_2_Se QDs and GO-GSH-ZnS/Ag_2_Se QDs nanocomposite.

| **Sample** | **C %** | **O%** | **S%** | **Zn%** | **Se%** | **Ag%** | **Au%** |
| --- | --- | --- | --- | --- | --- | --- | --- |
| QDs | 18.54 | 3.54 | 12.34 | 37.10 | 12.75 | 15.72 | - |
| QDs-GO | 40.34 | 10.79 | 5.89 | 27.73 | 4.17 | 11.08 | - |
| QDs-GO-CTAB-AuNPs | 42.55 | 12.84 | 5.08 | 18.49 | 6.49 | 11.70 | 2.85 |

**Table S2.** DLS hydrodynamic size and ZP values for GO, QDs, CTAB-AuNPs and the strep-B-QDs-GO-CTAB-AuNP biosensor probe.

| **Sample** | **DLS (nm)** | **ZP (mV)** |
| --- | --- | --- |
| GO | 528.0±78.7 | -14.3±3.4 |
| QDs | 363.0±77.3 | -7.5±3.9 |
| CTAB-AuNPs | 2.0±0.6 | 32.2±16.1 |
| strep-B-QDs-GO-CTAB-AuNP | 298.5±32.1 | -3.4±6.4 |

**References**

1. Gupta B, Kumar N, Panda K, Kanan V, Joshi S, Visoly-Fisher I (2017) Role of oxygen functional groups in reduced graphene oxide for lubrication. J Sci Rep-UK 7:45030
2. Beck G, Jank J (2008) Negative and linear positive magnetoresistance effect in silver-rich silver selenide. Solid State Sci. 10:776-789
3. Adegoke O, Forbes PBC (2016) _L_-Cysteine-capped core/shell/shell quantum dot–graphene oxide nanocomposite fluorescence probe for polycyclic aromatic hydrocarbon detection. Talanta 146:780–788
4. Adegoke O, McKenzie C, Nic Daeid N (2019) Multi-shaped cationic gold nanoparticle-L-cysteine-ZnSeS quantum dots hybrid nanozyme as an intrinsic peroxidase mimic for the rapid colorimetric detection of cocaine. Sensor Actuat B-Chem 287:416–427
5. Murray JB, Al-Shora HI (1978) Stability of cocaine in aqueous solution. J Clin Pharm 3:1-6
6. Isenschmid DS, Levine BS, Caplan YH (1989) A comprehensive study of the stability of cocaine and its metabolites. J Anal Toxicol 13:250:256.
